# Indacaterol provides 24-hour bronchodilation in COPD: a placebo-controlled blinded comparison with tiotropium

**DOI:** 10.1186/1465-9921-11-135

**Published:** 2010-10-05

**Authors:** Claus Vogelmeier, David Ramos-Barbon, Damon Jack, Simon Piggott, Roger Owen, Mark Higgins, Benjamin Kramer

**Affiliations:** 1 Universitätsklinikum Gießen und Marburg, Standort Marburg, Baldingerstraße, D-35043, Marburg, Germany; 2 Respiratory Department and Instituto de Investigación Biomédica de A Coruña (INIBIC), Complexo Hospitalario Universitario A Coruña, 15006, A Coruña, Spain; 3 Novartis Horsham Research Centre, Wimblehurst Road, Horsham, West Sussex RH12 5AB, UK; 4 Novartis Pharmaceuticals Inc., One Health Plaza, East Hanover, NJ 07936-1080, USA

## Abstract

**Background:**

Indacaterol is a novel, inhaled, once-daily, ultra-long-acting β_2_-agonist for the treatment of chronic obstructive pulmonary disease (COPD). This randomized, double-blind study compared the bronchodilator efficacy of indacaterol with that of placebo and tiotropium in patients with moderate-to-severe COPD.

**Methods:**

In an incomplete-block, multi-dose, three-period, crossover design, patients received three of the following four treatments: indacaterol 150 μg, indacaterol 300 μg, tiotropium 18 μg and placebo, each once-daily for 14 days. Each treatment period was separated by a 14-day washout. Study drug was supplied daily by blinded, third party study personnel to maintain blinding of patients and investigators. The primary efficacy variable was trough forced expiratory volume in one second (FEV_1_) at 24 h post-dose after 14 days. The study was powered to demonstrate non-inferiority of indacaterol to tiotropium for this variable.

**Results:**

A total of 169 patients were randomized (mean age 65 years); 153 (90.5%) completed. Trough FEV_1 _after 14 days with indacaterol 150 μg and 300 μg was statistically and clinically superior to placebo, with differences (95% CI) of 170 (120-220) and 150 (100-200) mL respectively (both p < 0.001). For this endpoint, both doses of indacaterol not only met the criterion for non-inferiority compared with tiotropium, but also achieved numerically higher values, with differences versus tiotropium of 40 and 30 mL for indacaterol 150 and 300 μg, respectively. At 5 min post-dose on Day 1, the mean FEV_1 _for both indacaterol doses was significantly higher than placebo (by 120 and 130 mL for indacaterol 150 and 300 μg, respectively; p < 0.001) and tiotropium (by 80 mL for both doses; p < 0.001). Adverse events were reported by similar proportions of patients: 31.4%, 29.5%, 28.3% and 28.5% for indacaterol 150 μg and 300 μg, tiotropium and placebo treatments, respectively.

**Conclusions:**

Once-daily indacaterol provided clinically and statistically significant 24-h bronchodilation. Indacaterol was at least as effective as tiotropium, with a faster onset of action (within 5 min) on the first day of dosing. Indacaterol should prove useful in patients with moderate-to-severe COPD, for whom treatment with one or more classes of long-acting bronchodilator is recommended.

**Trial registration:**

ClinicalTrials.gov: NCT00615459, EudraCT number: 2007-004071-19

## Background

According to the Global Initiative for Chronic Obstructive Lung Disease (GOLD), inhaled bronchodilators, including β_2_-agonists and anticholinergics, are central to the symptomatic management of chronic obstructive pulmonary disease (COPD) [1]. Currently available inhaled long-acting β_2_-agonists (LABAs), such as salmeterol and formoterol, provide bronchodilation for approximately 12 h at recommended doses, and hence are administered twice daily [2,3]. Tiotropium, the only currently available long-acting anticholinergic, has a duration of action of 24 h with once-daily dosing, and is effective in the long-term maintenance bronchodilator treatment of COPD [4-6]. Once-daily dosing of tiotropium has been shown to improve a range of clinical outcomes and exacerbations in patients with COPD compared with twice-daily LABAs and the anticholinergic ipratropium four times daily [5,7-9]. However, COPD remains a chronic disabling condition and additional therapeutic options are needed to achieve optimal disease management. Furthermore, patients' compliance with treatment could be improved if regimens are simplified by reducing the dosing frequency - especially given that the high incidence of comorbidities means that many patients with COPD require polypharmacy [10].

Indacaterol is a novel, inhaled, once-daily ultra LABA for the treatment of COPD [11]. Indacaterol has shown good overall safety and tolerability in clinical studies of up to 1-year duration with 24-h bronchodilator efficacy on once-daily dosing [12-17].

Two previous clinical trials have compared indacaterol with tiotropium, but due to the unavailability of a matching placebo, tiotropium was administered open-label [13,15]. The first study was a 7-day placebo-controlled dose ranging study, with an 8-day open-label tiotropium extension [13]. The second study was a 26-week pivotal study, in which, compared with open-label tiotropium, indacaterol 150 and 300 μg significantly increased the 24-h post-dose (trough) forced expiratory volume in 1 s (FEV_1_) after 12 weeks (primary endpoint) by 50 and 40 mL, respectively (both *p *≤ 0.01 vs tiotropium), with one or both indacaterol doses also significantly improving dyspnea, use of rescue medication and health status compared with tiotropium at most timepoints [15]. The present study was designed to complement these two earlier studies, by comparing the 24-h spirometry profile of indacaterol 150 and 300 μg once-daily with that of placebo and blinded tiotropium.

## Methods

This was a multinational, randomized, double-blind, double-dummy, placebo-controlled, multi-dose, Phase III, crossover study in patients with moderate-to-severe COPD. The study was conducted in accordance with the Declaration of Helsinki (1989) and local applicable laws and regulations. Institutional Review Board or Independent Ethics Committee approval was obtained for each participating study center. All participants provided informed written consent prior to taking part in the study.

### Study population

Male and female patients aged ≥40 years with moderate-to-severe COPD (GOLD 2006), a smoking history of at least 10 pack-years (current or previous smokers), post-bronchodilator FEV_1 _≥30% but <80% of the predicted normal value, and post-bronchodilator FEV_1_/forced vital capacity (FVC) <70% were included in the study.

Patients were excluded from the study if they had been hospitalized for a COPD exacerbation in the 6 weeks prior to screening or during the run-in period; had a history of asthma; or had concomitant pulmonary disease or a significant unstable cardiovascular or metabolic comorbidity.

### Study design and treatments

The study comprised a pre-screening visit, a 14-day screening period, followed by three 14-day treatment periods, each of which was separated by a 14-day washout. At the pre-screening visit, patients' ongoing COPD medications were reviewed and, if necessary, adjusted to exclude prohibited COPD therapies. On completion of the screening period, eligible patients were randomized using a validated automated system to receive three of the four treatments (with a different treatment in each treatment period), each once-daily. An incomplete-block design was selected (with three treatment periods rather than four) to reduce the overall burden on patients, and to increase the likelihood of patients completing the study (given the complexity in delivering the third-party blinded study medication - see below). Due to the use of different inhalers to deliver the study drugs, patients were randomized not only to a treatment sequence, but also to a sequence of inhalers. Indacaterol 150 or 300 μg was delivered via single-dose dry powder inhaler (SDDPI); tiotropium 18 μg was delivered via the manufacturer's proprietary single-dose dry powder inhalation device (HandiHaler^®^). Study drugs were inhaled between 06:00 h and 10:00 h on each day throughout the treatment periods.

Indacaterol and its matching placebo were made identical in appearance and were dispensed in such a manner so as to make them indistinguishable to patients and all blinded study personnel. As an exact physical match to tiotropium was not available, full blinding was achieved by third-party blinding procedures. These procedures were as follows: study drug was prepared and provided to the patient each morning, either at home or in the clinic, by persons who were independent of the other clinical trial processes (referred to as 'independent study blinding coordinators' or 'ISBCs') to preserve the integrity of the blind. Two ISBCs were required for each daily study drug administration to each individual patient. The first (unblinded) ISBC (who had no contact whatsoever with the patient) prepared the study drugs and devices, maintained patient, investigator and the second ISBC blinding and ensured that the patients strictly adhered to their allocated drug sequence. The second (blinded) ISBC provided the patient with the prepared study drug and devices and monitored administration of the drug by patients and also ensured that the blinding was maintained throughout. Both ISBCs completed the Third Party Blinding Log for every drug administration, in order to evidence that the blinding procedure was strictly followed.

### Concomitant medication

Allowable concurrent COPD therapies included the use of inhaled corticosteroids (ICS), provided the regimen had been stabilized for at least 1 month prior to the screening visit. Patients taking fixed-dose combinations of ICS and LABAs were switched to equivalent ICS monotherapy at a dose and dosage regimen maintained for the duration of the study. The following medications could not be used after the screening visit (except as study medication): long- or short-acting anticholinergic agents, long- or short-acting β_2_-agonists, xanthine derivatives, and parenteral or oral corticosteroids. Salbutamol was the only rescue medication permitted throughout the study, although not within 6 h prior to the start of each visit.

### Assessments and outcomes

All clinic visits started in the morning. In addition to the assessments during the screening visits, serial spirometry was performed on Day 1 and Day 14 of each treatment period, at 50 and 15 min pre-dose and at 5, 15 and 30 min and 1, 2, 4, 8, 10, 12 and 14 h post-dose. Spirometry was also assessed on Day 2 and Day 15 of each treatment period at 23 h 10 min and 23 h 45 min post-dose (based on the time of study drug administration on the previous day) to enable trough values of FEV_1 _to be determined. All spirometry evaluations were performed according to American Thoracic Society/European Respiratory Society standards [18].

Adverse events (AEs) and serious AEs were recorded, along with their severity, duration and relationship to study drug. Other safety assessments included: urinalysis; regular monitoring of hematology, blood chemistry (including serum potassium and blood glucose) and vital signs; and assessment of corrected QT interval (QTc).

The primary objective of the study was to determine whether indacaterol was superior to placebo as assessed by trough FEV_1 _after 14 days of treatment, with trough FEV_1 _defined as the mean of FEV_1 _measurements at 23 h 10 min and 23 h 45 min post-dose. The key secondary objective was a non-inferiority comparison between indacaterol and tiotropium for this endpoint (and if achieved, to then test for superiority). Other efficacy variables included trough FEV_1 _after the first dose, and FEV_1 _measurements at individual timepoints after the first dose and on Day 14 in each treatment period.

### Sample size calculation and statistical analysis

The study was powered for the key secondary objective, the non-inferiority comparison of indacaterol versus tiotropium for trough FEV_1 _after 14 days, where a non-inferiority margin of 55 mL based on a Cochrane review [19] was adopted. An advantage of 30 mL for indacaterol over tiotropium was assumed and a standard deviation of 220 mL for the difference between repeated measures on the same patient (based on information from previous studies). Taking account of the incomplete block nature of the design, 126 evaluable patients would provide a power of 90% for a one-sided test at the 1.25% significance level (half the usual alpha level to adjust for multiplicity). Allowing for a dropout rate of 15%, a total of 148 patients were planned to be randomized into this study. This number of patients would give 99% power for the primary endpoint, assuming a minimum clinically important difference (MCID) of 120 mL.

All efficacy variables, including the primary efficacy variable, were analyzed for the modified intent-to-treat (mITT) population comprising all randomized patients who received at least one dose of study drug. The non-inferiority comparison between indacaterol and tiotropium for trough FEV_1 _after 14 days was analyzed for the per-protocol population, which included all patients in the mITT population who had no major protocol deviations. The safety population included all patients who received at least one dose of study drug. Patients were analyzed according to treatment received.

An analysis of covariance model was used to analyze the primary endpoint and included terms for treatment, period, patient and period baseline value (pre-dose FEV_1 _on Day 1 of each treatment period), with results presented as least squares means, i.e., means adjusted for the covariates in the model. To allow for multiplicity, a Bonferroni adjustment was applied to maintain the overall Type I error rate at 5%. A similar model was used to analyze the secondary endpoints (with the non-inferiority and superiority comparisons between indacaterol and tiotropium also controlled for multiplicity).

## Results

### Patient disposition, demographics and baseline characteristics

A total of 211 patients were screened, 169 were randomized, and 153 (90.5%) completed. The most common reason for premature discontinuation was adverse events (n = 5), followed by administrative problems (4), abnormal test procedure results (3), withdrawal of consent (2), and unsatisfactory therapeutic effect (1). One patient was lost to follow up. Demographic and baseline characteristics of patients are summarized in Table 1.

**Table 1:** Demographics and baseline characteristics (safety population)

Characteristics	Patients (N = 167)
Age (years), mean (SD)	64.5 (7.92)
Sex, n (%)	
Male	128 (76.6)
Female	39 (23.4)
Race, n (%)	
Caucasian	165 (98.8)
Others	2 (1.2)
BMI (kg/m^2^), mean (SD)	26.8 (4.71)
Duration of COPD (years), mean (SD)	9.1 (7.91)
Smoking history, n (%)	
Ex-smoker	95 (56.9)
Current smoker	72 (43.1)
Number of pack-years, mean (SD)	43.1 (19.62)
Post-bronchodilator FEV_1 _(% predicted), mean (SD)	56.7 (13.58)
Post-bronchodilator FEV_1_/FVC (%), mean (SD)	50.1 (10.04)
FEV_1 _reversibility (% increase), mean (SD)	14.3 (12.26)

SD = standard deviation; BMI = body mass index; FEV_1 _= forced expiratory volume in 1s; FVC = forced vital capacity; pack-years = total years of smoking multiplied by cigarette packs smoked per day.

### Efficacy

**Figure 1:**
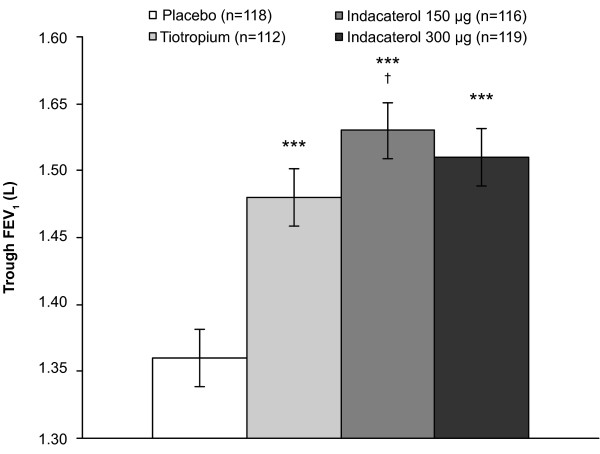
**24-h post-dose (trough) FEV_1 _(L) after 14 days of treatment (mITT population)**. Data are LSM ± SE. ***p < 0.001 vs placebo; ^†^p = 0.043 vs tiotropium. FEV_1_, forced expiratory volume in 1 s

For the primary endpoint (24-h post-dose [trough] FEV_1 _after 14 days of treatment), treatment with both doses of indacaterol resulted in statistically superior improvements compared with placebo, with least squares mean (LSM) treatment-placebo differences of 170 and 150 mL for the 150 and 300 μg doses that exceeded the 120 mL prespecified MCID (Table 2 and Figure 1). For this endpoint, both doses of indacaterol not only met the criterion for non-inferiority compared with tiotropium, but also achieved numerically higher values, with differences versus tiotropium of 40 and 30 mL for indacaterol 150 and 300 μg, respectively, in the per-protocol population. The p-value for the statistical comparison of superiority between indacaterol 150 μg and tiotropium was 0.043, with a LSM treatment difference of 50 mL (mITT population), although this comparison did not meet the formal requirement for superiority (which was for the 97.5% confidence interval to be entirely above zero).

**Table 2:** Treatment contrasts of trough FEV_1 _(L) after 1 and 14 days of treatment (mITT population)

Treatment contrast	Treatment difference		
	**LS mean ± SE**	**97.5% CI^**	**p-value**
Day 14			
Indacaterol 150 μg - Placebo	0.17 ± 0.023	(0.12,0.22)	< 0.001*
Indacaterol 300 μg - Placebo	0.15 ± 0.023	(0.10,0.20)	< 0.001*
Tiotropium - Placebo	0.12 ± 0.023	(0.07,0.17)	< 0.001*
Indacaterol 150 μg - Tiotropium	0.05 ± 0.023	(-0.01,0.10)	0.043
Indacaterol 300 μg - Tiotropium	0.03 ± 0.023	(-0.03,0.08)	0.249
	**LS mean ± SE**	**95% CI**	**p-value**
Day 1			
Indacaterol 150 μg - Placebo	0.10 ± 0.021	(0.06,0.15)	<0.001*
Indacaterol 300 μg - Placebo	0.13 ± 0.021	(0.09,0.17)	<0.001*
Tiotropium - Placebo	0.10 ± 0.021	(0.06,0.14)	<0.001*
Indacaterol 150 μg - Tiotropium	0.01 ± 0.021	(-0.04,0.05)	0.772
Indacaterol 300 μg - Tiotropium	0.03 ± 0.021	(-0.01,0.08)	0.101

LS = least squares, SE = standard error of the mean, CI = confidence interval

^ 95% confidence interval for the comparison tiotropium minus placebo.

*Statistically significant comparison (two-sided p-value < 0.05).

For trough FEV_1 _after the first dose, both doses of indacaterol were again statistically superior to placebo, with the 300 μg dose exceeding the 120 mL MCID (LSM treatment-placebo difference 130 mL, *p *< 0.001; Table 2). The mean trough FEV_1 _values after treatment with both indacaterol 150 and 300 μg were numerically higher than with tiotropium, by 10 and 30 mL, respectively.

**Figure 2:**
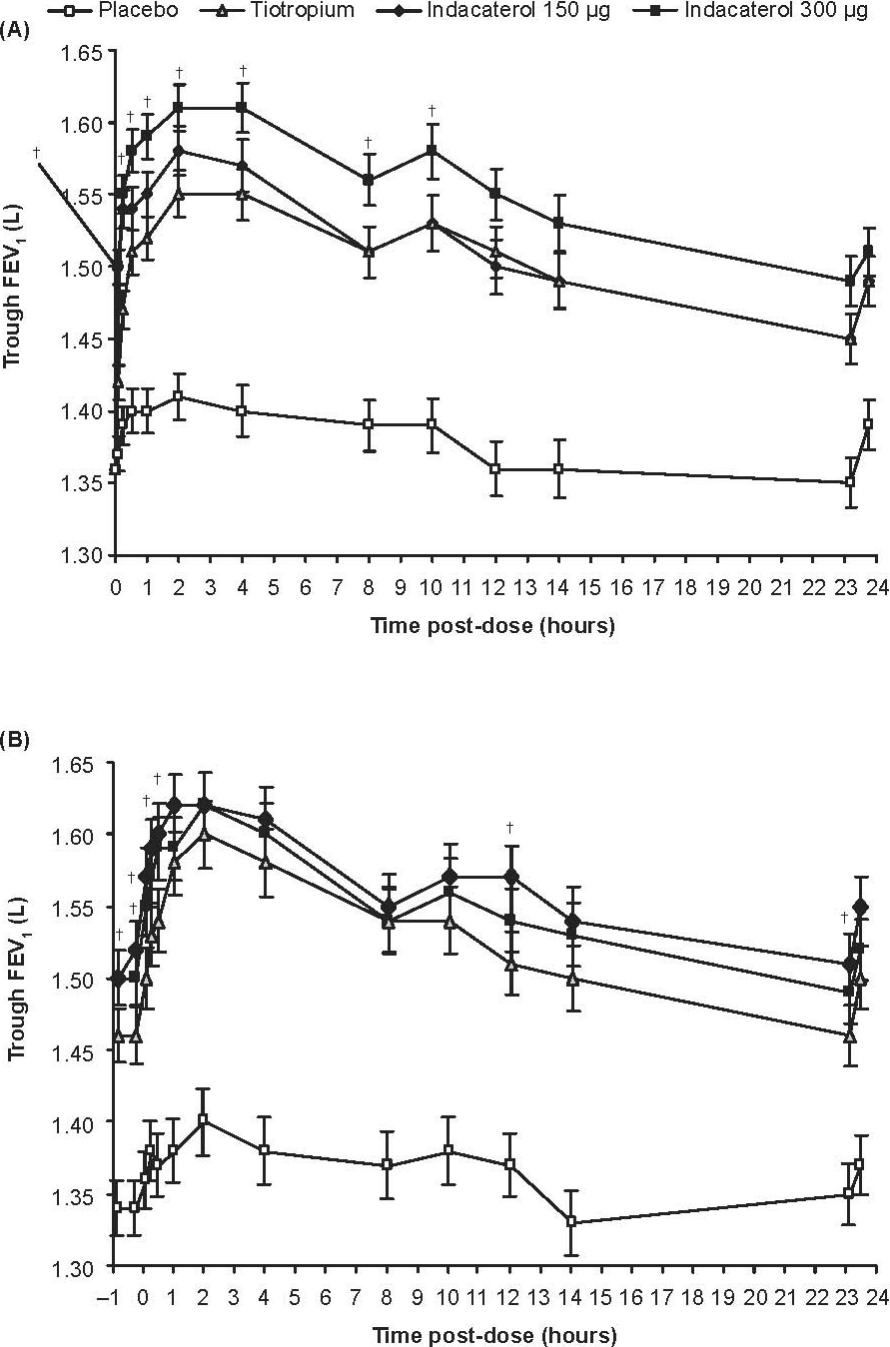
**24-h profile of least squares means of FEV_1 _on Day 1 (A) and 14 (B) (mITT population)**. A) Data are LSM ± SE. p < 0.001 for indacaterol (150 and 300 μg) vs placebo at each timepoint, p < 0.001 for indacaterol, 150 μg vs tiotropium at 5 and 15 min, ^†^p < 0.05 for indacaterol 300 μg vs tiotropium, p < 0.05 for tiotropium vs placebo at each timepoint. B) Data are LSM ± SE. p < 0.001 for indacaterol (150 and 300 μg) and tiotropium vs placebo at each timepoint, ^†^p < 0.05 for indacaterol 150 μg vs tiotropium at -50 to 30 min, 12 h and 23 h 10 min, p < 0.05 for indacaterol 300 μg vs tiotropium at 5 min

At all time points on both the first day and after 14 days of treatment, all active treatments resulted in statistically significantly greater FEV_1 _results compared with placebo (Figure 2). The LSM FEV_1 _for indacaterol was numerically larger than for tiotropium at all timepoints for the 300 μg dose, and at a majority of timepoints for the 150 μg dose.

Both indacaterol doses had a fast onset of action on Day 1, providing clinically relevant treatment-placebo differences in LSM FEV_1 _at 5 min post-dose of 120 and 130 mL for indacaterol 150 and 300 μg, respectively (*p *< 0.001 for both), compared with 50 mL for tiotropium (*p *< 0.004). At this timepoint, treatment with both indacaterol doses resulted in statistically superior FEV_1 _to tiotropium (LSM differences of 80 mL for both indacaterol doses, *p *< 0.001).

### Safety

The overall incidence of AEs was similar across all treatments, and were predominantly mild or moderate in severity (Table 3). The most frequent AEs were cough (indacaterol 150 μg, 6.8%; indacaterol 300 μg, 4.9%; tiotropium, 2.5%; placebo, 2.4%), COPD worsening (5.1, 3.3, 8.3, 8.9%) and nasopharyngitis (3.4, 7.4, 4.2, 4.9%). None of the AEs leading to study drug discontinuation were suspected to be study-drug-related.

**Table 3:** Adverse events overall and by primary system organ class (safety population)

	Indacaterol 150 μg N = 118 n (%)	Indacaterol 300 μg N = 122 n (%)	Tiotropium N = 120 n (%)	Placebo N = 123 n (%)
Patients with any AE(s)	37 (31.4)	36 (29.5)	34 (28.3)	35 (28.5)
**MedDRA primary system organ class**				
Respiratory, thoracic & mediastinal disorders	17 (14.4)	14 (11.5)	15 (12.5)	15 (12.2)
Infections & infestations	7 (5.9)	14 (11.5)	10 (8.3)	10 (8.1)
Musculoskeletal & connective tissue disorders	6 (5.1)	6 (4.9)	2 (1.7)	9 (7.3)
Nervous system disorders	4 (3.4)	3 (2.5)	5 (4.2)	3 (2.4)
Gastrointestinal disorders	2 (1.7)	3 (2.5)	8 (6.7)	3 (2.4)
Injury, poisoning & procedural complications	2 (1.7)	0	1 (0.8)	1 (0.8)
Metabolism & nutrition disorders	2 (1.7)	2 (1.6)	0	0
Blood & lymphatic system disorders	1 (0.8)	1 (0.8)	1 (0.8)	0
Cardiac disorders	1 (0.8)	1 (0.8)	0 (0.0)	2 (1.6)
General disorders & administration site conditions	1 (0.8)	2 (1.6)	3 (2.5)	3 (2.4)
Investigations	1 (0.8)	0	1 (0.8)	0
Psychiatric disorders	1 (0.8)	0	1 (0.8)	0
Ear & labyrinth disorders	0	0	0	1 (0.8)
Neoplasms benign, malignant & unspecified (including cysts and polyps)	0	0	1 (0.8)	0
Renal & urinary disorders	0	2 (1.6)	1 (0.8)	0
Skin & subcutaneous tissue disorders	0	2 (1.6)	1 (0.8)	0
Vascular disorders	0	2 (1.6)	1 (0.8)	4 (3.3)

Primary system organ classes are sorted in descending order of frequency in the indacaterol 150 μg treatment

SAEs were reported in one patient while taking indacaterol 300 μg (COPD exacerbation), two patients while taking indacaterol 150 μg (both COPD exacerbations), four patients while taking tiotropium (three reported as COPD exacerbation and one reported as cerebrovascular accident), and one patient while taking placebo (COPD exacerbation). None of these were suspected to be related to study medication by the investigators. There were no deaths during the study, although one patient died during the 30-day follow-up period due to an acute myocardial infarction and infection; this was not suspected to be study-drug-related (the patient received indacaterol 300 μg in the first treatment period, indacaterol 150 μg in the second, and tiotropium in the third).

There were no clinically notable serum potassium values (defined as a post-baseline value <3.0 mmol/L) during treatment with either of the indacaterol doses. One patient experienced a clinically notable potassium value during treatment with tiotropium. The incidence of clinically notable blood glucose levels (defined as a post-baseline value of >9.99 mmol/L) during treatment with indacaterol 150 μg was 8.5% (10/118), 7.4% (9/122) during treatment with indacaterol 300 μg, 2.5% (3/120) during treatment with tiotropium and 7.3% (9/123) during placebo treatment.

No patient had an abnormally high pulse rate (>130 bpm, or ≥120 bpm and increase from baseline ≥15 bpm). The proportion of patients with newly occurring or worsening QTc interval (Fridericia's) >450 ms (males) or >470 ms (females) was lower during treatment with indacaterol 150 μg (2.5%) compared with indacaterol 300 μg (4.9%), tiotropium (5.0%) and placebo (4.1%). No patient had a maximum post-baseline increase in Fridericia's QTc of >60 ms or an absolute value >500 ms.

## Discussion

This randomized, double-blind study compared the 24-h spirometry profile of indacaterol 150 and 300 μg once-daily with that of tiotropium 18 μg once-daily and placebo in patients with moderate-to-severe COPD. The primary efficacy analysis showed that once-daily indacaterol 150 μg and 300 μg provided clinically relevant improvements in 24-h post-dose (trough) FEV_1 _after 14 days of treatment. The improvement versus placebo in bronchodilation with both indacaterol doses was not only higher than the 100 mL criterion described by Donohue [20] as a difference that COPD patients can perceive but also exceeded the prespecified clinically relevant difference of 120 mL, and moreover was above the range (100-140 mL) that has been proposed as a range of values for a minimal clinically important difference [21]. These results are consistent with those observed in long-term studies [16,17,22], which also confirm that there is no loss in efficacy with once-daily dosing of indacaterol for up to a year.

In the current study, indacaterol provided a 30-50 mL higher bronchodilator effect than tiotropium in terms of trough FEV_1 _after 14 days of treatment. Although there is no consensus for a clinically relevant threshold for differences between active treatments, in other studies tiotropium was associated with improvements in trough FEV_1 _over both salmeterol (52 mL, *p *< 0.01) and formoterol (42 mL, *p *< 0.05) [5,23]; the further improvements over tiotropium of a similar magnitude achieved with indacaterol may be considered at least noteworthy. Further, the efficacy results of this study support the results of the previous 26-week pivotal study conducted by Donohue et al [15], in which tiotropium was administered on an open-label basis. The magnitude of treatment difference between indacaterol and tiotropium after 2 weeks in the present study (50 and 30 mL for indacaterol 150 and 300 μg, respectively) was similar to that observed after 12 weeks of treatment in the pivotal study (50 and 40 mL, respectively) [15]. Therefore, the results from the current blinded study validate the results of the earlier pivotal study. In the present study, indacaterol demonstrated a fast onset of action after the first dose with FEV_1 _improvements that were statistically superior to both placebo and tiotropium at the first post-dose timepoint (5 min), with differences from placebo at or above the prespecified 120 mL minimum clinically important difference. This result is also consistent with the findings of the pivotal study, in which at 5 min following the first dose both indacaterol doses resulted in statistically superior FEV_1 _to tiotropium (*p *< 0.001) [15].

Given that an exact physical match to tiotropium was not available, a very difficult third-party blinded approach - probably the first of its kind - was employed in this study. This required two study personnel, independent of any other study procedures, to visit each patient daily during the treatment periods, with one of these personnel blinded to the identity of the study medication then handing the prepared inhalers to the patient. It is of note that despite this, a low premature discontinuation rate was observed in this study. A crossover design (rather than a parallel-group design) was chosen because the within-patient variability in FEV_1 _was expected to be less than between-patient variability with each patient acting as their own control. An incomplete-block, rather than a complete-block, crossover design was adopted (with three periods) to reduce the overall burden on patients. For the incomplete-block design the within-patient variability is higher than that of a complete block design, and this therefore required a higher number of patients to be recruited. The 14-day time point was selected as primary endpoint in the present study, because previous studies have shown that indacaterol reaches pharmacodynamic steady-state prior to this time [12,17], as does tiotropium [24], with the bronchodilator efficacy observed after 2 weeks similar to that observed after 12 weeks for both drugs [15]. The duration of two weeks for washout was also sufficient to minimize the possibility of carry-over effects of both indacaterol and tiotropium, and the length of this washout period increased the practicability of the study by permitting each treatment period to start on the same day of the week. Further, the difference in trough FEV_1 _between tiotropium and placebo observed in this study was similar to that reported previously [4].

Overall, all treatments in this study (including placebo) had good safety and tolerability profiles. The overall incidence of AEs was comparable across all treatment groups. Most AEs were mild or moderate in severity, and the majority were related to COPD and respiratory symptoms - as expected in this patient population. Although the most common AE in patients treated with indacaterol was cough, these events were mild or moderate in severity and were not associated with discontinuation from the study. Class-related side effects of inhaled β_2_-agonists (e.g., hyperglycemia, hypokalemia or prolonged QTc interval) were observed at a similar incidence with both indacaterol doses as with placebo.

## Conclusions

Indacaterol at doses of both 150 and 300 μg given once daily, resulted in clinically relevant 24-h bronchodilation with a fast onset of action in patients with moderate-to-severe COPD, and demonstrated a good overall safety and tolerability profile. The bronchodilator efficacy of indacaterol appears to be at least comparable with that of tiotropium, with a faster onset of action. Indacaterol may prove useful in patients with moderate-to-severe COPD, for whom treatment with one or more classes of long-acting bronchodilator is recommended.

## Competing interests

This study was funded by Novartis Pharma AG, Basel, Switzerland. Damon Jack, Simon Piggott, Roger Owen, Mark Higgins, Benjamin Kramer are employees of Novartis. Claus Vogelmeier gave presentations at symposia sponsored by (in alphabetical order) Altana, Astra Zeneca, Aventis, Bayer, Boehringer, Chiesi, GlaxoSmithKline, Merck Darmstadt, Novartis, Pfizer, Talecris, and received fees for consulting from (in alphabetical order) Altana, Astra Zeneca, Bayer, Boehringer, GlaxoSmithKline, Janssen-Cilag, Talecris. David Ramos-Barbon was a speaker at conferences sponsored by AstraZeneca, Merck Sharp&Dohme, GlaxoSmithKline, Pfizer and Esteve and received advisory board fees from GlaxoSmithKline.

## Authors' contributions

DJ, SP, RO, MH and BK (as employees of the study sponsor, Novartis) contributed to the design, analysis and interpretation of the study, and oversaw its conduct. CV and DRB were involved in the collection of data. All authors contributed equally to the development of the manuscript, and approved the final version for submission.
